# The salivary gland proteome of root-galling grape phylloxera (*Daktulosphaira vitifoliae* Fitch) feeding on *Vitis* spp.

**DOI:** 10.1371/journal.pone.0225881

**Published:** 2019-12-17

**Authors:** Markus W. Eitle, James C. Carolan, Michaela Griesser, Astrid Forneck

**Affiliations:** 1 University of Natural Resources and Life Sciences, Department of Crop Sciences, Institute of Viticulture and Pomology, Vienna, Austria; 2 Department of Biology, Maynooth University, Maynooth, Co. Kildare, Ireland; Onderstepoort Veterinary Institute, SOUTH AFRICA

## Abstract

The successful parasitisation of a plant by a phytophagous insect is dependent on the delivery of effector molecules into the host. Sedentary gall forming insects, such as grape phylloxera *(Daktulosphaira vitifoliae* Fitch, *Phylloxeridae*), secrete multiple effectors into host plant tissues that alter or modulate the cellular and molecular environment to the benefit of the insect. The identification and characterisation of effector proteins will provide insight into the host-phylloxera interaction specifically the gall-induction processes and potential mechanisms of plant resistance. Using proteomic mass spectrometry and *in-silico* secretory prediction, 420 putative effectors were determined from the salivary glands or the root-feeding *D*. *vitifoliae* larvae reared on Teleki 5C (*V*. *berlandieri* x *V*. *riparia*). Among them, 170 conserved effectors were shared between *D*. *vitifoliae* and fourteen phytophagous insect species. Quantitative RT-PCR analysis of five conserved effector candidates (protein disulfide-isomerase, peroxidoredoxin, peroxidase and a carboxypeptidase) revealed that their gene expression decreased, when larvae were starved for 24 h, supporting their assignment as effector molecules. The *D*. *vitifoliae* effectors identified here represent a functionally diverse group, comprising both conserved and unique proteins that provide new insight into the *D*. *vitifoliae*–*Vitis* spp. interaction and the potential mechanisms by which *D*. *vitifoliae* establishes the feeding site, suppresses plant defences and modulates nutrient uptake.

## Introduction

The ability of phytophagous insects to feed from, or reproduce on plant hosts is dependent on the efficient modulation or evasion of plant defence systems. These defences may be constitutive or induced and can involve complex recognition and response systems that result in the release of defensive substances, targeted self-destruction of cells or even the attraction of predators to contend with the invading insect. Insects and particularly those that engage the plant host on cellular level, attempt to overcome these defences through a variety of strategies including the delivery of bioactive substances into the plant cellular environment [1]. Many of these substances, commonly referred to as effectors, are delivered via the insect saliva directly on- or into the host tissue and ultimately determine whether the prospected interaction is compatible or incompatible. Effectors of phytophagous insects are generally small proteins or molecules that alter host cell structures and/or biological functions once they are exposed to a suitable plant tissue. Thereby single effectors may interact with several plant protein networks and modulate various host physiological traits simultaneously [2, 3].

Identifying the effector repertoire of phytophagous insect species is essential to understand the underlying host-parasite interaction and potentially leads to the development of innovative pest control strategies reducing reliance on agrochemical application [4, 5]. The identification of effector molecules within plant tissues is challenging due to their low concentration and short ephemerality due to metabolic processing. An alternative strategy is to characterise the effector-enriched salivary gland tissue, which previously resulted in the discovery of multiple insect effector proteins released by aphids [6–8], hessian flies [9], planthoppers [10, 11], whiteflies [12, 13] and thrips [14]. Significant progress in protein-based mass spectrometry and next generation sequencing technologies (e.g. RNAseq) facilitated the large-scale identification of putative insect effectors lists within insect salivary glands or saliva [15–21]. Comparative *In-silico* analyses of predicted effector sets across phloem-feeding aphid species identified both conserved effectors used as part of shared infestation strategies aimed to modulate common host defensive and cellular processes and unique effectors employed to establish and maintain compatible interactions in a host-specific manner [22–24].

Historically grape phylloxera (*Daktulosphaira vitifoliae* Fitch; *Phylloxeridae*) was introduced from North America into Europe in the late 19^th^ century with devastating economic consequences for the wine industry at the time. The sedentary insect infests the *Vitis spp*. root system by the formation of root gall tissues, thereby inducing changes in the water, mineral and assimilate transport affecting the host vine physiology and vigor [25–27]. In addition *D*. *vitifoliae* infestation promotes secondary soil-borne infections harming the root system of field-grown vines [28, 29]. The majority of commercial *Vitis* rootstocks tolerate minor phylloxera populations on the root system [30], however reports of phylloxera-caused root damages in vineyards increase worldwide [31–38]. Root gall formation by *D*. *vitifoliae* involves the regulation of various host plant physiological traits including the induction of structural root gall growth [39–43], interference with plant defence mechanisms [44–47] and alteration of systemic nutrient allocation [41, 48, 49].

Insect salivary glands are the principal organs for the production of effectors essential for the establishment and maintenance of compatible host-parasite interactions. In this study we aim to identify and characterise effector proteins of root-galling *D*. *vitifoliae*, a major pest of *Vitis* spp. hypothesising that salivary glands dissected from root-feeding *D*. *vitifoliae* larvae are enriched with effector proteins of diverse functional effector groups involved in the modulation of host physiological pathways and required for insect feeding.

## Material and methods

### Plant and insect material

Dormant single eye cuttings of the rootstock Teleki 5C (*V*. *berlandieri* x *V*. *riparia*, clone Gm 6–52) were propagated in Jiffy-7 pots (40 mm; Jiffy Products International AS, Norway) under controlled greenhouse conditions. In total 48 rooted plants were transplanted in four growth containers (38 x 28 x 20 cm) containing a mixed substrate of perlite and foamed clay (1:1). The growth containers were located in a climate chamber set to (25°C, 60% rH and 16 h photoperiod) and fertigated weekly (0.25‰ Ferty 3 MEGA) in quarantine conditions [50, 51]. Two hundred eggs of a *D*. *vitifoliae* single founder lineage were placed adjacent to the roots of each plant growth container for hatching and asexual reproduction.

### Salivary gland collection

After 50 days feeding *D*. *vitifoliae* L3 larvae [52] were carefully from root galls and submerged in ice-cold phosphate buffered saline (PBS) with pH 7.0 (VWR, Radnor, USA) containing a protease inhibitor cocktail (0.2 mM, Complete Mini, Roche, Basel, CH). For each biological sample the salivary glands of 100 larvae were dissected under the binocular using two metallic needles and immediately snap-frozen in liquid N_2_. The salivary gland samples for the qRT-PCR analyses were collected likewise, but from L3 larvae submerged in a PBS buffer containing RNAlater (Qiagen, Hilden, DE). For the qRT-PCR analyses salivary glands were dissected from feeding (SG0) and starving *D*. *vitifoliae* larvae (SG24). For the starvation treatment, feeding L3 larvae were gently removed from the root galls and kept without plant contact on moist filter paper in isolated petri dishes for 24 h at 25±3°C in the dark simulating equal environmental conditions. For each treatment four or three biological samples were collected and stored at -80°C for the subsequent proteomic and transcriptomic analyses, respectively.

### Protein extraction and purification

Salivary glands were homogenized with a motorised pestle in 150 μl lysis buffer (7 M urea, 2 M thiourea supplemented with a protease inhibitor cocktail, Roche, Complete Mini). Samples were briefly sonicated and centrifuged at 9000 g for 5 min to pellet the cellular debris. The supernatant was removed and purified using the 2D clean-up Kit (GE Heath Care, Chicago, USA) following the manufacturer’s instructions (procedure A) and the resulting protein pellet was resuspended in 50 μl of 6 M urea, 2 M thiourea, 0.1 M Tris-HCl, pH 8.0 and quantified using the Qubit Protein assay Kit (Thermo Scientific, Waltham, USA). 20 μl of this resuspension was removed for protein digestion and 50 mM ammonium bicarbonate was added to each sample. Proteins were reduced with 0.5 M dithiothreitol (DTT) at 56°C for 20 min and alkylated with 0.55 M iodoacetamide (IAA) at room temperature for 15 min, in the dark. 1 μl of a 1% w/v solution of Protease Max Surfactant Trypsin Enhancer (Promega) and 1 μg of Sequence Grade Trypsin (Promega) was added and the protein/trypsin mixture was incubated at 37°C for 18 hours. Digestion was terminated by adding 1 μl of 100% trifluoroacetic acid (Sigma Aldrich) and incubation at room temperature for 10 min. Samples were centrifuged for 10 min at 13,000 × g and a volume equivalent to 40 μg of pre-digested protein was removed and purified for mass spectrometry using ZipTips (Merck Millipore, Burlington USA) following the manufacturer’s instructions and the resulting eluant was concentrated with a SpeedyVac (Savant DNA120, Thermo Scientific Waltham, USA) and resuspended in acetonitrile (2% v/v); TFA (0.05% v/v) buffer.

### Mass spectrometry and functional annotation

A volume equivalent to 1 μg of tryptic peptides was loaded onto a QExactive high resolution accurate mass spectrometer (Thermo Scientific Waltham, USA) connected to a Dionex Ultimate 3000 chromatography system (RSLCnano, Thermo Scientific Waltham, USA). The peptides were separated by a 2% to 40% gradient of acetonitrile on a Biobasic C18 PicofritTM column (100 mm length, 75 mm ID), using a 120 minute reverse-phase gradient at a flow rate of 250 nL min-1. All data were acquired with the mass spectrometer operating in automatic data dependent switching mode. A full MS scan at 140,000 resolution and a range of 300–1700 m/z was followed by an MS/MS scan, resolution 17,500 and a range of 200–2000 m/z, selecting the 15 most intense ions prior to MS/MS.

Protein identification and LFQ normalisation of MS/MS data was performed using MaxQuant v1.5.6.5 (http://www.maxquant.org) to correlate data against the predicted protein set derived from the *D*. *vitifoliae* genome version 3.2 [53] produced and provided by the International Aphid Genomic Consortium (IAGC) (bipaa.genouest.org/is/aphidbase) and a contaminant sequence set provided by MaxQuant. The following search parameters were used: first search peptide tolerance of 20 ppm, second search peptide tolerance 4.5 ppm with cysteine carbamidomethylation as a fixed modification and N-acetylation of protein and oxidation of methionine as variable modifications and a maximum of two missed cleavage sites allowed. False Discovery Rates (FDR) were set to 1% for both peptides and proteins and the FDR was estimated following searches against a target-decoy database. Perseus v.1.5.5.3 (www.maxquant.org/) was used for data analysis, processing and visualisation. The data matrix was first filtered for the removal of contaminants and peptides identified by site. LFQ intensity values were log_2_ transformed, ranked on their average MS intensities and proteins not found in three of the four replicates were omitted from the analysis.

Functional annotation of the identified proteins and the assignment of gene ontology (GO) terms was conducted using Blast2GO v. 2.5.0 [54]. BlastP searches were conducted against the NCBI nr database (as of 12.11.2018) reporting matches with a HSP cut-off value ≥ 33, BLAST hits with an E≤10^−3^ value and > 90% sequence similarity were reported. GO terms for all identified proteins were categorized by molecular function (MF), biological process (BP) and cellular component (CC) to determine the functional component of the salivary gland proteome. Signal secretion peptides were identified using a combined *In-silico* pipeline consisting of: TMHMM Server 2 [55], PredGPI [56], SignalP Server 5.0 for *Eukarya* [57] and SecretomeP Server 2.0 with NN-score > 0.6 [58]. Local BlastP analyses against published salivary effector candidates of fourteen phytophagous insect species [11, 15, 16, 19, 24, 59–62] were performed with NCBI’s Blast+ tool. Effectors having an e-value < e^-50^ and a bit score >100 were considered as orthologous matches for the effector candidates of *D*. *vitifoliae*.

### RNA extraction and qRT-PCR analysis

RNA was extracted using the NucleoSpin RNA XS kit (Macherey-Nagel, Düren, Germany) following manufacturer’s instructions yielding 100–250 ng total RNA per sample. Subsequent reverse transcription (Sensiscript RT kit, Qiagen, Hilden, Germany) and normalisation were conducted using 50 ng of cDNA. Out of six candidate genes, actin A1 (DV3000100) was chosen as a reference gene after optimisation tests [63] following the Normfinder procedure [64]. The primer pairs were designed based on the *D*. *vitifoliae* genome version 3.2 using the NCBI primer tool software (Table 1). Quantitative RT-PCR analyses were performed using the Rotor-Gene Cycler Q (Qiagen, Hilden, Germany) employing KAPA SYBR FAST qPCR Universal (Kapa Biosystems, Wilmington, US) as detector agent in technical duplicates (total reaction vol. 12 μl with 2 μl cDNA (1:7)). Primer efficiencies were determined by conducting standard curves with four step template dilutions. Cycling conditions were: one cycle for 5 min at 95°C, 45 cycles for 8 sec at 95°C, 20 sec at 60°C, 15 sec at 72°C, 3 sec at 75°C [48]. Relative gene expression levels were calculated using the EasyqpcR package (bioconductor) in *R*. Statistics were performed with Independent T tests (p < 0.05) in SPSS (IBM, v24) comparing the gene expression levels in salivary glands dissected from feeding larvae (SG0) versus starving larvae at 24 h (SG24).

**Table 1:** Analysed DvEffector genes and primers used for qRT-PCR.

Gene accession ID (Dv 3.2)		Annotation	Forward sequence	Reverse sequence	R^2^	Primer efficiency
			(5'-3')	(5'-3')		
**Reference gene**						
DV3000100	**actA1**	Actin A1	TGGGGTGGTAGTGGTGATGA	ACACGTCGGATGTAAACGACA	0,981	1,10
**DvEffectors matched with effectors of *Acyrthosiphon pisum***						
DV3009058.1	**Dv1**	Protein disulfide-isomerase A3	TCATGGCTTAGTTGGACATCGT	TTACGCCAGTAGTTGGTTCCT	0,997	0,98
DV3001833	**Dv2**	Peroxiredoxin-2	AAACCAGCCCCAGATTGGAA	TTGAACGCCAATATCTCAGTTGG	0,999	0,94
DV3017719	**Dv3**	Venom serine carboxypeptidase-like	CAGAGCTGCGTGCAAAGTGA	ACCAAAGCAAGACAGACGCA	0,993	0,93
DV3010201	**Dv4**	Peroxiredoxin-2	TGTTTTAGTGGAACCTGATGGTGT	AGCCTGAACAAGCCGTAAGAC	0,997	1,09
DV3014676	**Dv5**	Peroxidase	TCTGCGACAACAGTGACGAT	TGTGGAGCAGCATGACCATA	0,997	0,96
**False positives**						
DV3014415	**Fp1**	Muscle-specific 20-like	ATTGAATATGGCGTTCCTGATGTAG	AGGGCCTAAAAATGGACCTTTCC	0,993	1,02
DV3008102	**Fp2**	Myofilin isoform a	CAGATCCATACCATTCTGAGCCT	TCGGGCAGAGGTAGAGGTTT	0,993	0,96
DV3011193	**Fp3**	Serine arginine repetitive matrix 1 isoform X1	AGTTCAACGTCCAGCGGAAA	ACCGGGAATCACTTTCTTCGT	0,989	0,99

The presented genes coded for proteins identified within the salivary gland proteome of *D*. *vitifoliae* larvae feeding on root galls of Teleki 5C. The gene accession IDs refer to the *D*. *vitifoliae* genome v 3.2 provided by the International Aphid Genomic Consortium (IAGC). ‘Dv 1–5’ refers to five conserved DvEffectors, whereas ‘Fp1-3’ to false positives functionally not associated to *D*. *vitifoliae* feeding. The gene annotation corresponds to the *A*. *pisum* effector ortholog.

## Results

### Assessment and description of the salivary gland proteome of *D*. *vitifoliae*

**Fig 1:**
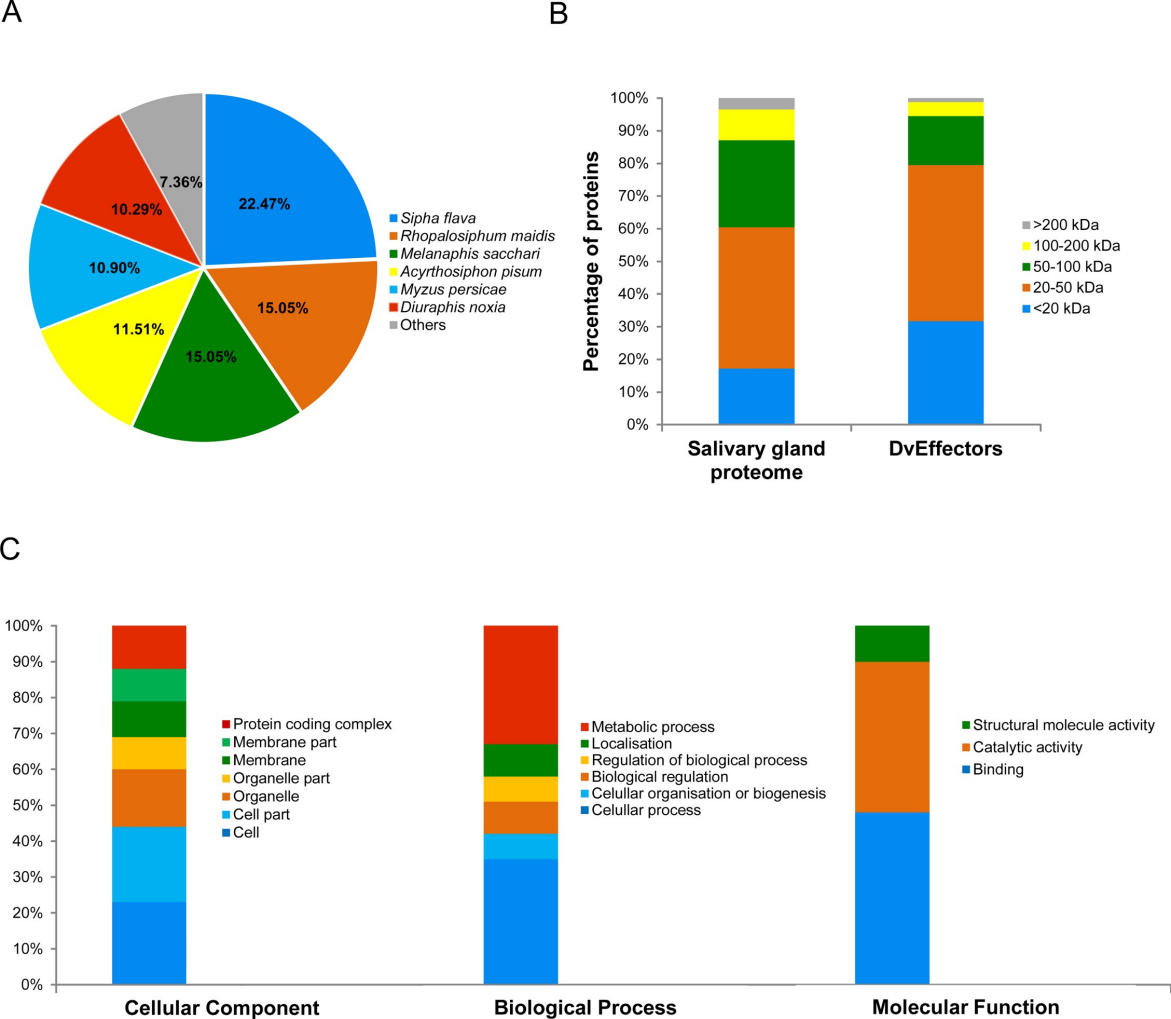
Characterisation of the salivary gland proteome. Gene ontology characterisation of the salivary gland proteome of *D*. *vitifoliae* larvae feeding on root galls of Teleki 5C (*V*. *berlandieri* x *V*. *riparia*). **A)** Protein top blast hit analysis ranked by species; **B)** Molecular weight distribution [kDa] of 1511 proteins of the salivary gland proteome and the secreted DvEffectors subset (420 proteins); **C)** GO enrichment analysis conducted with Blast2Go.

The dissection of the salivary glands from 100 feeding *D*. *vitifoliae* larvae, subsequent protein extraction and purification yielded 3.5–6.0 μg of total protein. In total 19,901 unique peptides representing 1511 proteins were identified (hereafter the salivary gland proteome) with log_2_ normalised LFQ intensities ranging between 21.6–35.9 (S1 Table). Comparative blast searches conducted with Blast2Go (as of 12.11.2018) revealed high similarities between the salivary gland proteome of *D*. *vitifoliae* and the aphid species. In total 92.64% of the identified proteins had top blast hits with proteins from: *Sipha flava* (22.47%), *Rhopalosiphum maidis* (15.05%), *Melanaphis sacchari* (15.05%), *Acyrthosiphon pisum* (11. 51%), *Myzus persicae* (10.90%) and *Diuraphis noxi*a (10.29%) (Fig 1A). The majority of the identified salivary gland proteins, did not exceed 100 kDa in weight (87.09%) (Fig 1B). The smallest identified protein DV3009370 was annotated as cytochrome b-c1 complex subunit 9 (6.91 kDa, 59 AA), whereas the biggest protein DV3004164 was an annotated titin isoform X2 (1630.90 kDa, 14427 AA) (S1 Table). Of the top 100 most abundant proteins, determined by relative ranking based on average intensities, 36 were associated with structural components including contractile muscles, the cytoskeleton and the extracellular matrix. In addition, numerous primary metabolic enzymes (7) and mitochondrial associated proteins (14) were abundantly represented. To explore the functional composition of the salivary gland proteome in more detail, a Gene Ontology (GO) term analysis was conducted and terms were grouped into the categories: cellular component, molecular function and biological process (Fig 1C). For the cellular component category, 44% of the annotated proteins was associated to ‘cell’ or ‘cell part’, 25% to ‘organelle’ or ‘organelle part’, 19% to ‘membrane’ or ‘membrane part’ and 12% to ‘protein-containing complexes’. Within the biological process category the two most abundant groups were ‘cellular process’ 35% and ‘metabolic process’ 42%. The protein distribution within the category molecular function resulted in 42% of proteins with ‘binding’ properties, 40% with ‘catalytic activity’ and 10% with ‘structural molecule activity’ (Fig 1C).

### DvEffectors identified within the salivary glands of *D*. *vitifoliae*

A combined *In-silico* pipeline was employed to resolve proteins that are secreted with high probability among the *D*. *vitifoliae* salivary gland proteome (Table 2). The first step of the presented *In-silico* pipeline involved the screening for amino acid based structural retaining signals/motifs. Proteins with one or more transmembrane helices (TMHMMs) and/or glycosylphosphatidylinositol-anchors (GPI-anchors) were excluded as effectors due to their high probability of being retained by intra- and extracellular membranes. The remaining proteins without structural retaining signals were analysed for their likelihood to be secreted via classical (SignalP) or non-classical leaderless (SecretomeP, NN-score > 0.6) secretion pathway. Applying these criteria 420 *D*. *vitifoliae* proteins (27.80%) were detected to be secreted (Table 2) and therefore represented the putative *D*. *vitifoliae* effector set named ‘DvEffectors’ (S2 Table). The subsequent molecular weight analysis of the filtered DvEffectors showed a higher proportion of smaller proteins in comparison to the overall salivary gland proteome. Most of the identified DvEffectors (94.52%) did not exceed 100 kDa (Fig 1B).

**Table 2:** Protein secretion pipeline applied to the *D*. *vitifoliae* salivary gland proteome.

**Secretory prediction pipeline**	**Protein number**	**Percentage**
Identified proteins	1511	100,00%
TMHMMs	240	15,88%
GPI-anchors	23	1,52%
**Proteins without structural retaining signals**	**1259**	**82,32%**
Classical secretion pathway	151	10,19%
Non-classical secretion pathway	354	23,43%
**Secreted proteins**	**420**	**27,80%**

The secretion pipeline used to identify secreted proteins within the salivary gland proteome of *D*. *vitifoliae* larvae feeding on root galls of Teleki 5C (*V*. *berlandieri* x *V*. *riparia*). The online tools TMHMM Server 2.0 (TMHMMs), PredGPI (GPI-anchors), SignalP Server 5.0 (classical secretion pathway) and SecretomeP Server 2.0 (non-classical secretion pathway) were used in a stepwise fashion to determine the likelihood of proteins to be secreted.

**Fig 2:**
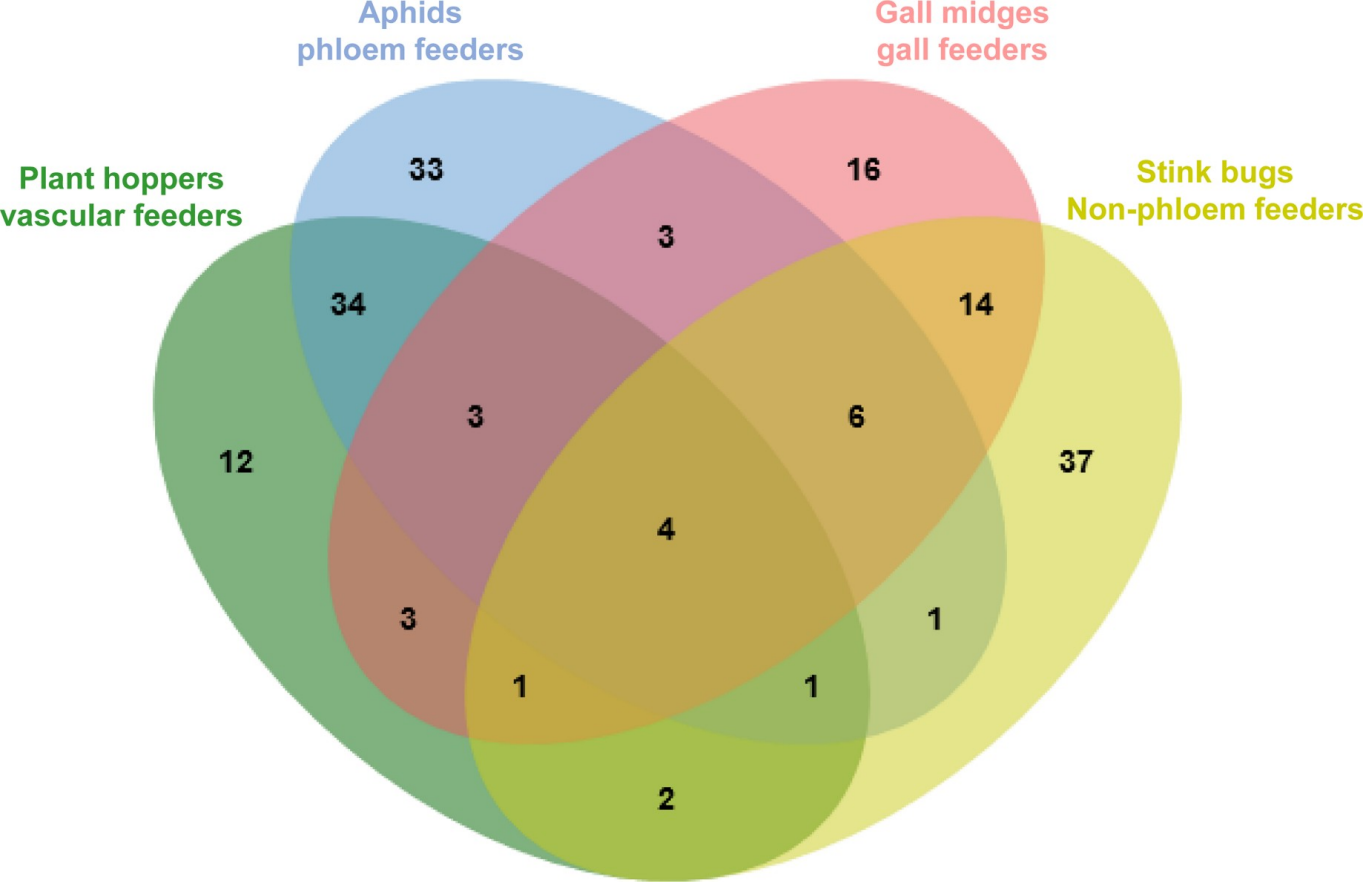
Conserved DvEffectors with phytophagous insects. A summary of the BlastP search results of the DvEffectors against salivary gland proteins of phytophagous insects with different feeding behaviour: Plant hoppers–vascular feeders (green): *Nephotettix cincticeps* [11], *Nilaparvata lugens*, *Sogatella frucifera*, *Laodelphax striatellus* [59]; Aphids—phloem feeders (blue): *Acyrthosiphon pisum* [15, 16], *Myzus persicae*, *Rhopalosiphum padi*, *Myzus cerasi* [24], *Sitobion avenae* [19]; Gall midges—gall feeders (rosa): *Mayetiola destructor* [61], *Mayetiola avenae*, *Mayetiola hordei* [60] and Stink bugs—non-phloem feeders (yellow): *Halyomorpha halys* and *Nezara viridula* [62]. Numbers within the circles indicated the ortholog matches (-value < 1.0 e^-50^ and a bit score >100) between DvEffectors and the salivary gland proteins of at least one insect species within the insect feeding category.

To identify potentially conserved effectors shared between *D*. *vitifoliae* and other phytophagous insect species, BlastP searches were conducted against salivary proteins of vascular (xylem and phloem) feeding plant hoppers: *Nephotettix cincticeps* [11], *Nilaparvata lugens*, *Sogatella frucifera*, *Laodelphax striatellus* [59]; phloem feeding aphids: *Acyrthosiphon pisum* [15, 16], *Myzus persicae*, *Rhopalosiphum padi*, *Myzus cerasi* [24], *Sitobion avenae* [19]; gall feeding gall midges: *Mayetiola destructor* [61], *Mayetiola avenae*, *Mayetiola hordei* [60] and non-phloem feeding stink bugs: *Halyomorpha halys* and *Nezara viridula* [62]. The conducted analysis (e-value < 1.0 e^-50^ and bit score >100) identified 170 conserved DvEffectors shared with salivary proteins of at least one analysed insect species (Fig 2, S3 Table). As one example 42 DvEffectors matched with predicted salivary effectors of the pea aphid *Acyrthosiphon pisum* (S4 Table, Table 3), being the species with most effector matches. Among them we identified numerous functional effector annotations such as esterases, glucose dehydrogenases, heat shock proteins, lysosomal alpha-mannosidases, maltases, peroxidases, peroxidoredoxins, protein disulfide-isomerases and carboxypeptidases (Table 3).

**Table 3:** DvEffector matches with effectors of *Acyrthosiphon pisum*.

Functional annotation	*Acyrthosiphon pisum* effector	*Daktulosphaira vitifoliae* ortholog	E-value	Bit score
**Esterases**	XP_001947304.2	DV3004001.2	0.00E+00	569.
		DV3013307.2	4.24E-97	302.
**Glucose dehydrogenase**	XP_001943395.1	DV3014986	0.00E+00	905.
**Heat shock protein**	NP_001156420.1	DV3001393	0.00E+00	1020
**Lysosomal alpha-mannosidase**	XP_008187164.1	DV3016723	0.00E+00	1390
**Maltase**	XP_001943582.2	DV3005150	0.00E+00	555.
**Peroxidase**	XP_001951217.2	DV3006190	0.00E+00	1206
		DV3014676*..	1.71E-168	493.
**Peroxidoredoxins**	XP_001949571.1	DV3001833*..	1.01E-156	430.
		DV3010201*..	1.07E-91	265.
		DV3001185.2	2.63E-92	265.
**Protein disulfide-isomerase**	XP_001950406.1	DV3009058.1*	0.00E+00	871.
	XP_008183165.1	DV3000226	0.00E+00	755.
**Carboxypeptidases**	XP_001943316.2	DV3017719*..	2.82E-152	434.
	XP_008183303.1	DV3004740.2	1.57E-70	239.

Blast results between the DvEffectors set against effector lists of *Acyrthosiphon pisum* [15, 16] using the BlastP software of NCBI were presented. The match quality is determined by the e-value and bit score. The functional annotations are taken from the effector list of *Acyrthosiphon pisum*. Asterisks and bold protein IDs highlight effector candidates (Dv1-5) employed for the following qRT-PCR analysis.

### Biosynthesis of *D*. *vitifoliae* effector candidates

**Fig 3:**
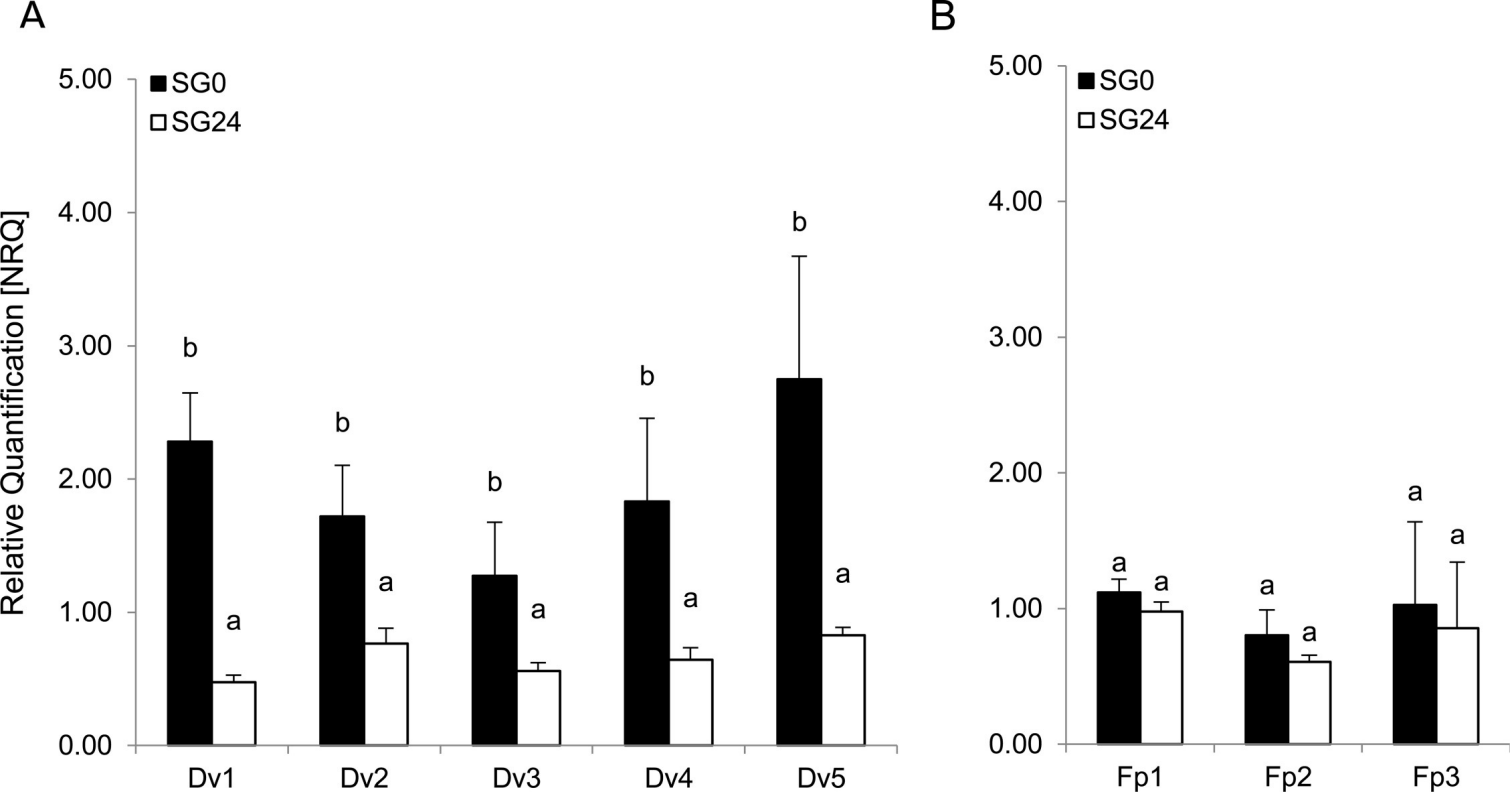
Relative gene expression levels of *D*. *vitifoliae* effector genes. Expression levels [NRQ] of genes coding for A) conserved effectors of *D*. *vitifoliae* and *A*. *pisum* (Dv1-5) and B) false positives detected within the salivary gland proteome of *D*. *vitifoliae* but not associated to feeding (Fp1-3). RNA was extracted from salivary glands dissected from of feeding (SG0) and starving larvae (SG24). ActinA1 (DV3000100) was used as reference gene. Error bars indicate standard deviations of three independent biological replicates each consisting of salivary glands dissected from 100 individuals. Minor letters refer to significant differences obtained by Independent T-tests (p < 0.05).

To increase the effector status of the predicted DvEffector candidates we challenged the hypothesis that the expression of the underlying DvEffector genes decreased when not required for feeding (= starved). In total five conserved effectors orthologs of *Acyrthosiphon pisum* (Dv1-5, Table 1) were chosen for a qRT-PCR analysis using RNA samples extracted from the salivary glands of feeding (SG0) and 24 h starving (SG24) *D*. *vitifoliae* larvae. In addition, three control metabolism genes, unrelated to insect feeding, were chosen (Fp1-3, Table 1) hypothesising that they their relative expression levels in salivary gland tissue remains unaffected by larval starvation. The results of the qRT-PCR analysis (Fig 3) showed significant gene expression levels for all tested candidate genes (Dv1-5 and Fp1-3) in salivary gland tissue of feeding and starving *D*. *vitifoliae* larvae (Fig 3A). Among them the gene expression level of Dv5 annotated as peroxidase was ranked as the highest with 2.75 NRQ in SG0. Comparing the SG0 and SG24 treatments, the analysis showed that the gene expression levels of all five DvEffectors decreased significantly, when insects were starved for 24 h without plant contact (Fig 3A), whereas the gene expression levels of Fp1-3 were statistically not affected by larval starvation (Fig 3B).

## Discussion

We present the first proteomic profile of salivary glands dissected from a root galling single founder lineage of *D*. *vitifoliae* feeding on root galls of Teleki 5C (*V*. *berlandieri* x *V*. *riparia*). Based on the salivary gland proteome and a subsequent *In-silico* secretory prediction 420 putative ‘DvEffectors’ were identified. Protein matches with salivary effector candidates of other phytophagous insects resulted in the identification of 170, conserved effectors. Quantitative RT-PCR analyses of five DvEffectors exemplarily complemented their potential roles as effectors since their expression within the salivary glands decreased by larval starvation.

### The salivary gland proteome of *D*. *vitifoliae* is similar to aphids

In total 92.64% of identified *D*. *vitifoliae* proteins had a top blast hits with six aphid species (Fig 1A). Phylloxerids, in particular *D*. *vitifoliae*, are often described as ‘aphid-like’ or close aphid relatives because of their evolutionary relationship, similar modes of life and shared environmental niches [65, 66]. In fact *Aphidoidea* and *Phylloxeroidea* are suggested to share a common ancestor about 250 million years ago [67]. Recent genomic and transcriptomic studies confirm the phylogenetic proximity of *D*. *vitifoliae* and *Aphidoidea* [53, 68, 69]. Our results underlined these findings by showing a high degree of consistency between the proteomic profiles of *D*. *vitifoliae* salivary glands and aphids. Furthermore we showed a strong similarity in the effector profiles of root galling belowground *D*. *vitifoliae* and leaf feeding aphids as evidenced by the identification of conserved effector proteins (Fig 2 and S3 Table). In fact generalist and specialist insect species utilise conserved ‘core’ effectors involved in general plant infestation strategies [24, 70].

### Conserved DvEffector groups

In total 420 DvEffectors of a *D*. *vitifoliae* single founder lineage feeding on root galls were identified based on their salivary gland proteome, among them 170 conserved effectors secreted by root feeding *D*. *vitifoliae* and other phytophagous insect species (Fig 2, S3 Table). Among them we detected numerous conserved DvEffectors associated to plant defence suppression such as peroxidoredoxins (DV3001185.2, DV3010201, DV3001833), peroxidases (DV3014676, DV3013176), superoxide dismutase (DV3003945) and serine protease (DV3001393) independently of the insect feeding category (S4 Table). For phloem feeders (aphids and plant hoppers) we found orthologous effector candidates potentially involved in stylet sheath formation (disulfide isomerases (DV3004088.2, DV3005250) and mucins (DV3010687.2, DV3001963) and DvEffector orthologs associated to cuticle functions proteins (e.g. DV3000905.2, DV3004090.2, DV3006395). Interestingly gall midges and stink bugs have an increased number of proteins associated to ribosomal processes (e.g. DV3005082.2, DV3007542.2, DV3007763.2). In the following we associated selected conserved effectors (Table 3) to functional effector groups and discuss their role for the compatible *D*. *vitifoliae* -*Vitis* spp. root interaction.

The first group is composed by DvEffectors involved in insect feeding site establishment. Protein disulfide isomerases (PDI) are commonly detected within insect salivary glands [16, 61, 71, 72]. Their abundancy across insect species with piercing-sucking mouthparts is explained by their catalytic role in the cross-linkage of cysteine bonds between mucin molecules essential for the stylet sheath formation [73, 74]. Other references suggest that phytophagous parasites secrete PDIs to detoxify host defensive reactive oxygen species [75, 76]. For *D*. *vitifoliae* we report the combined secretion of PDIs and mucins presuming their essential role for feeding site establishment by facilitating the stylet penetration into the root cortex and maintaining its mobility within the root gall tissue during insect feeding. Esterases and mannosidases, secreted by invading insects and phytopathogens, represent two groups of digestive enzymes assisting in the degradation of plant cell walls [77, 78]. Mobile *D*. *vitifoliae* larvae penetrate the cortexes of root tips or secondary lignified roots in order to establish the root gall feeding site [39, 41]. The secretion of enzymatic effectors contributing to the destabilisation of root cuticular layers and cell walls might facilitate the stylet penetration into the root cortex tissue. In addition increased cell wall flexibility could favour cell tissue expansion of root galls as shown exemplarily for the induction host expansin genes [43].

The second group of DvEffectors is composed by proteins primarily involved in the suppression of host plant defences. Glucose dehydrogenases (GDH) are among the most abundant proteins effectors detected within aphid saliva [79, 80]. Based on their structural similarity to glucose oxidases, GDHs are suggested to interfere with host plant defence mechanisms such as alterations of the salicylic and jasmonic acid signalling cascades, detoxification of reactive oxygen species, ethylene or other defensive compounds [81, 82], all of which might be part of the root infestation strategy of *D*. *vitifoliae*. Peroxidases and peroxidoredoxins catalyse the reduction of hydrogen peroxide (H_2_O_2_) known to be associated to incompatible host-parasite interactions such as hypersensitive cell death responses (HR) [83, 84]. Through the external delivery of detoxifying and scavenging enzymes into the root gall feeding site, *D*. *vitifoliae* might encounter the accumulation of defensive secondary metabolites to protect itself and/or to sustain the biological functionality of the feeding tissue; thus maintaining the compatible interaction despite activated host plant defences. Heat shock proteins (HSP) are evolutionary conserved key regulators known to interfere with stress signal cascades [85, 86]. In plants HSPs trigger the activation of host innate immunity traits against invading insect species [87], which would suggest a rather negative impact on the compatible *D*. *vitifoliae*–*Vitis* spp. interaction. On the opposite site HSPs trigger protective traits against a range of abiotic stresses [88], which would favour the sustainability of *D*. *vitifoliae* root galls in the soil. For instance in *D*. *vitifoliae* leaf galls HSPs are suggested to play a role for the suppression of host defences and the carbohydrate allocation [89].

*D*. *vitifoliae* feeds on the cell content withdrawn from parenchymal root cortex tissue marked by depleted levels of dissolved carbohydrates and elevated levels of free amino acids as compared to aphid species [90–92]. This implies the employment effectors assisting in and optimising the uptake of essential nutrients within the liquid diet. Maltases and GHDs are important enzymes for the endogenous sugar metabolism of insects [93, 94]. Both enzymes are previously detected in salivary glands of several aphids [16, 17, 19, 79, 80] thought to be involved in the extra oral digestion of carbohydrates increasing the digestibility of the sugar-rich phloem sap [14]. Root galls of *D*. *vitifoliae* contain abundant starch amounts due to redirected sucrose from the host plant’s primary metabolism [48]. It is not yet clear whether the accumulated starch serves as nutrient reservoir for the insect and its offspring or whether it represents a way to balance potentially phytotoxic sucrose levels. Nonetheless the secretion of sugar cleaving enzymes likely enables *D*. *vitifoliae* to exploit and feed from the accumulated carbohydrate reservoir within the root galls. Carboxypeptidases are involved in protein digestion processes aimed to provide peptides or amino acids for a balanced insect nutrition [95]. *D*. *vitifoliae* root galls and tuberosities contain elevated levels of essential amino acids such as glutamate, glutamic acid and asparagine and aspartic acid [41]. Secreted carboxypeptidases might be responsible for the protein or peptide cleavage explaining the accumulation of free amino acids in order to be dissolved and imbibed within the parenchymal sap by the insect. Additionally carboxypeptidases possess antimicrobial properties against bacterial and fungal pathogens [96, 97]. Alternatively carboxypeptidases may suppress feed-competitive or saprophytic microorganisms in the root tissue, which otherwise could contaminate the insect feed or degrade the host gall tissue [28].

### DvEffectors involved in structural root gall formation

Among the set of DvEffectors, 65 (15.48%) do not have a functional annotation (‘not annotated’ or ‘uncharacterized protein’) and might represent novel *D*. *vitifoliae-*specific or even biotype-specific candidates involved in the establishment of a compatible root interaction. In particular, the identity and function of insect effectors that modulate plant physiological pathways resulting in structural root gall formation are yet unknown. However, through comparisons to identified effectors in other phytophagous organisms the potential function of newly identified effectors can be made. Although the feeding sites of root galling nematode species such as *Heterodera* sp., *Globodera* sp. [98] and *Meloidogyne* sp. [99] are structurally different from *D*. *vitifoliae* root galls [39, 41], effectors involved in root galling could be expected to have evolutionary conserved functions [100]. Based on experimental evidence Hassan (et al. 2010) [101] characterised effectors of root-galling nematodes with stringent biological functions involved in cell wall degradation, suppression of host plant defences, feeding site initiation, development and maintenance. Comparing the nematode effectors associated with structural root gall formation with the DvEffectors, numerous proteins are common to both. These include calreticulins (DV3007536, DV3007818.2) and proteins associated to ubiquitination (DV3014649, DV3013030, DV3002279, DV3001602, DV3004034, DV3012664) as well as nuclear DNA/RNA modifications (e.g. DV3010515, DV3009903.2, DV3016166, DV3010607 DV3002380, DV3001693.2, DV3017446 DV3019764, DV3008705, DV3003913, DV3000194) and could be the effector candidates associated with root gall formation described for an insect species.

### Insect starvation supports effector status

In order to provide further evidence for the effector status of the identified DvEffector set, the biosynthesis of five selected effector candidates Dv1-5 (Table 1) was investigated on transcriptional level. A decrease in expression was observed for all five target genes in *D*. *vitifoliae* larvae that were removed from the gall and starved for 24 h (Fig 3). *D*. *vitifoliae* as a sedentary insect is in continuous contact with the root gall tissue and offers the possibility to assess temporarily well-defined starvation treatments [44, 48, 97]. When dealing with insect starvation, one key factor is the intensity and duration of the prospected treatment. A starvation regime that is too abrupt may affect overall insect metabolism and behaviour [102], whereas a targeted and mild starvation, as conducted in the present study, merely affects the metabolic processes related to insect feeding [103]. Here we show that the expression of effectors genes within the salivary glands of root-galling *D*. *vitifoliae* larvae decreased, when insects were starved for 24 h confirming the validity of our approach to provide improved characterisation and methodological complementation of the presented DvEffector set.

## Supporting information

S1 Table The proteomic profile of salivary glands dissected from *D*. *vitifoliae* L3 larvae feeding on root galls of Teleki 5C (*V*. *berlandieri* x *V*. *riparia*). Proteins IDs were based on the *D*. *vitifoliae* genome v. 3.2. Annotations and protein length were obtained by BlastP searches against the NCBI nr database (as of 12.11.2018).
(XLSX)

S2 Table DvEffectors candidates identified within salivary glands of *D*. *vitifoliae* L3 larvae feeding on root galls of Teleki 5C (*V*. *berlandieri* x *V*. *riparia*). Proteins IDs were based on the *D*. *vitifoliae* genome v. 3.2. Annotations and protein length were obtained by BlastP searches against the NCBI nr database (as of 12.11.2018).
(XLSX)

S3 Table BlastP results of conserved DvEffectors and salivary proteins of phytophagous insects. The first table provided information about the salivary gland protein sets used for the BlastP analysis referring to the feeding category, insect species and the literature reference. The second table presented conserved DvEffectors shared with plants hoppers (green), aphids (blue), gall midges (rosa) or stink bugs (yellow). Proteins IDs were based on the *D*. *vitifoliae* genome v. 3.2. (as of 12.11.2018).
(XLSX)

S4 Table BlastP results of conserved DvEffectors between root-galling *D*. *vitifoliae* and three aphid species. Proteins IDs were based on the *D*. *vitifoliae* genome v. 3.2. The table presented the BlastP results between the identified DvEffector set against effector lists of *Acyrthosiphon pisum* with a cut-off e-value < e^-50^ and bit score > 100. Protein annotations were taken from the effector lists of *Acyrthosiphon pisum* [15, 16].
(XLSX)
